# Vitamin D metabolic pathway genes polymorphisms and vitamin D levels in association with neonatal hyperbilirubinemia in China: a single-center retrospective cohort study

**DOI:** 10.1186/s12887-023-04086-y

**Published:** 2023-05-31

**Authors:** Weiwei Zhou, Ping Wang, Yanrui Bai, Ying Zhang, Jianbo Shu, Yang Liu

**Affiliations:** 1 Department of Neonatology, Tianjin Children’s Hospital/Tianjin University Children’s Hospital, No. 238 Longyan Road, Beichen District, Tianjin, 300134 China; 2 Graduate College, Tianjin Medical University, Tianjin, China; 3 Tianjin Pediatric Research Institute, Tianjin Children’s Hospital, Tianjin University Children’s Hospital, No. 238 Longyan Road, Beichen District, Tianjin, 300134 China; 4 Tianjin Key Laboratory of Birth Defects for Prevention and Treatment, Tianjin, China

**Keywords:** Neonatal hyperbilirubinemia, Vitamin D levels, Single nucleotide polymorphisms, Vitamin D metabolic pathway gene

## Abstract

**Background:**

Neonatal hyperbilirubinemia (NH) is a major cause of hospitalization after birth. Previous studies indicated that vitamin D deficiency might play an important role in NH susceptibility, but the results were controversial. Meanwhile, there has been limited description of the association between vitamin D related genes single nucleotide polymorphisms (SNP) and NH susceptibility. We aimed to investigate the vitamin D metabolic pathway genes polymorphisms and vitamin D levels with NH susceptibility.

**Methods:**

We retrospectively analyzed the clinical data, vitamin D levels and its metabolic pathway gene polymorphisms of 187 NH neonates and 149 controls at Tianjin Children’s Hospital/Tianjin University Children’s Hospital between April 2019 and August 2022. Vitamin D levels were measured by liquid chromatography-tandem mass spectrometry (LC-MS/MS) method, and the genetic polymorphism of *NADSYN1/DHCR7*, *GC*, *CYP2R1*, *CYP24A1* and *CYP27B1* was detected by high resolution melting (HRM) analysis.

**Results:**

The frequency of vitamin D deficiency (25(OH)D < 15 ng/mL) was significantly increased in the NH group compared to controls. TT genotype of rs12785878 and GT genotype of rs10877012 were protective factors of vitamin D deficiency and NH, and GT genotype and dominant model carriers of rs12785878 had a higher risk of severe NH than the GG genotype carriers (GT genotype: OR: 2.43; 95% CI: 1.22–4.86; *P* = 0.012, dominant model: OR: 1.97; 95% CI: 1.04–3.73; *P* = 0.037). *GC* gene haplotype was associated with vitamin D deficiency. No significant SNP-SNP and SNP-vitamin D levels interaction combinations were found.

**Conclusions:**

There were associations among NH, vitamin D deficiency and *NADSYN1/DHCR7* and *CYP27B1* polymorphisms, TT genotype of rs12785878 and GT genotype of rs10877012 could reduce the risk of vitamin D deficiency and NH. Furthermore, rs12785878 was significantly associated with severe NH.

**Supplementary Information:**

The online version contains supplementary material available at 10.1186/s12887-023-04086-y.

## Background

Neonatal hyperbilirubinemia (NH) is a common clinical phenomenon in newborns[1]. The condition from a metabolic imbalance between the production and elimination of bilirubin[2]. NH is generally benign and transient, but about one in ten neonates are likely to develop clinically severe hyperbilirubinemia[3]. Infants with severe NH may progress to bilirubin encephalopathy and kernicterus, associated with long-term neurodevelopmental impairments or even death[4]. The etiology of NH is complex, and risk factors for severe NH include genetic, maternal, perinatal, neonatal and other factors[3]. Besides, Asian neonates are at higher risk of hyperbilirubinemia[5]. Based on the crippling complications of NH and the huge population base of China, early recognition of high risk factors are particularly important in Chinese neonates, which will contribute to timely intervention and adequate management to reduce the occurrence of sequelae.

Vitamin D is a fat-soluble micronutrient and steroid hormone precursor that plays an essential role in human health. It regulates calcium homoeostasis and influences many physiological functions, including cell growth, proliferation, differentiation, apoptosis, immune function and inflammation[6]. Hyperbilirubinemia and vitamin D deficiency are issues of concern in neonates, and one step in both bilirubin and vitamin D synthesis occurs in the liver, although the metabolic pathways are different[7]. Therefore, it is speculated that vitamin D levels may be associated with hyperbilirubinemia in newborn. To our knowledge, only a few studies investigated the relationship between hyperbilirubinemia and vitamin D levels and the results are controversial[8–11].

Some single nucleotide polymorphisms (SNP) in the vitamin D metabolism genes including 7-dehydrocholesterol reductase, NAD synthetase 1 (*NADSYN1*/*DHCR7)*, vitamin D binding protein (*GC*), 25-hydroxylase (*CYP2R1*), 24-hydroxylase (*CYP24A1*) and 1-α-hydroxylase (*CYP27B1*), have been linked to 25(OH)D concentrations and several loci were identified by the genome-wide association studies (GWAS)[12–18]. These genes are mainly involved in the production, binding, transport, metablism and activation of vitamin D and the variants influence serum 25(OH)D levels. A few studies have focused on the relationship between hyperbilirubinemia and polymorphisms in vitamin D-related genes [19]. So far, few studies have examined the relationship among vitamin D levels, vitamin D metabolic pathway genes polymorphisms and hyperbilirubinemia and neonatal serum vitamin D. Given the high prevalence of NH and the importance of identifying its risk factors, understanding the relationship among these can play a vital role in the diagnosis and treatment of NH.

Thus, our study aimed to shed light on the association among serum 25(OH)D concentrations, 6 SNPs of 5 genes involved in vitamin D metabolism (*NADSYN1/DHCR7* rs12785878, *GC* rs4588 and rs7041, *CYP2R1* rs12794714, *CYP24A1* rs17216707 and *CYP27B1* rs10877012) and the risk of NH.

## Methods

### Study subjects

We retrospectively analyzed neonates admitted to Tianjin Children’s Hospital/Tianjin University Children’s Hospital between April 2019 and August 2022. Neonates meeting the following criteria were enrolled in the study: postnatal age of ≤ 14 days; term or near-term appropriate for gestational age (GA) neonates, as defined by birth weight (BW) ≥ 2000 g for ≥ 36 weeks GA or BW ≥ 2500 g for ≥ 35 weeks GA[20, 21]. Exclusion criteria were Rhesus Macacus (Rh) incompatibility, Glucose-6-Phosphate Dehydrogenase (G6PD) deficiency, sepsis, asphyxia, hypothyroidism, congenital malformations, cholestasis, congenital biliary atresia, or liver disease that would affect the total serum bilirubin (TSB) level.

336 newborns were recruited into this study, including the NH group (n = 187 cases) and control group (n = 149 cases). The diagnosis of NH is based on the guidelines of The Society of Pediatrics Chinese Medical Association published in 2010[22]. We divided the enrolled patients into the NH group whose TSB ≥ 75th percentile according to the guidelines mentioned above and the control group whose TSB < 75th percentile or without jaundice. The neonates of the NH group were divided into three groups based on the TSB levels: 76 cases in the mild group (≥ 75th percentile-phototherapy threshold), 93 cases in the moderate group (phototherapy threshold- exchange transfusion threshold), and 18 cases in the severe group (above the exchange transfusion threshold). The information recorded included days of age, gender, GA, BW, exclusive breastfeeding and maternal characteristics.

### Measurement of serum vitamin D levels

The concentrations of 25(OH)D were measured by liquid chromatography-tandem mass spectrometry (LC-MS/MS). 25 (OH) D levels were divided into deficiency (≤ 15 ng/ml), insufficient (> 15 ng/ml and ≤ 20 ng/ml), and sufficient (> 20 ng/ml)[23]. The season of blood collection was divided into summer/fall (June to November) and winter/spring (March to May and December to February), considering the duration of sunlight exposure.

### SNPs selection and genotyping

Six SNPs in five genes, which have been associated with serum vitamin D concentrations from GWAS studies and systematic reviews, were genotyped, including *NADSYN1/DHCR7* (rs12785878), *GC* (rs4588, rs7041), *CYP2R1* (rs12794714), *CYP24A1* (rs17216707) and *CYP27B1* (rs10877012)[12–18]. We used high resolution melting (HRM) for genotyping in this study, which is a high-efficiency and economical technology for mutation, polymorphism and epigenetic alteration detection in double-stranded DNAs [24]. Genomic DNA was isolated using the genomic DNA kit (Beijing ComWin Biotech) according to the manufacturer’s instructions and stored at -20 °C until further analysis.

The sequences of these six loci were provided by the SNP database of NCBI (https://www.ncbi.nlm.nih.gov/snp/?term). Furthermore, DNAMAN9 software and Primer-BLAST (https://www.ncbi.nlm.nih.gov/tools/primer-blast/index.cgi?LINK_LOC=BlastHome) were utilized to perform primer (Supplementary Table 1) design and specificity analysis. Then the PCR amplification was performed and the amplified products were sent to Sanger sequencing. The sequencing results were analyzed with Chromas2.6.4 sequence analysis software to obtain the required HRM reference samples. HRM assay was carried out by a LightCycler^®^480, and the melting curves were analyzed using LightCycler 480 Software 1.5. Four samples from each group were submitted for Sanger sequencing to confirm the accuracy of genotyping by HRM assay.

### Statistical analysis

In this study, IBM SPSS statistical software 26.0 was used for statistical analysis. The measurement data conforming to the normal distribution were expressed by the mean ± standard deviation (Mean ± SD), and the comparison between groups was analyzed by Student’s t-test or one-way ANOVA. The measurement data that did not conform to the normal distribution were expressed by the median (interquartile range) [Median (IQR)], and the comparison between groups was performed by the Nonparametric tests. Counting data were expressed in percentage (%) and were compared by χ^2^ test or Fisher’s exact test. The deviation of the Hardy-Weinberg equilibrium (HWE) for genotype frequencies was analyzed by χ^2^ test. Logistic regression was used to analyze the relationship among genotypes, 25 (OH) D levels and NH risk. Generalized multifactor dimensionality reduction (GMDR) was used to find out the potential interaction combinations between SNPs and environmental factors, including SNP-SNP synergy and SNP-vitamin D synergy effect. Linkage disequilibrium (LD) and haplotypebased case-control analysis were performed by Haploview 4.2 software[25]. And a *P*-value less than 0.05 was regarded as statistically significant.

## Results

### Demographic and Clinical Characteristics of the Study Subjects

The baseline characteristics of the study population are shown in Table 1. Cases and controls were comparable for age, GA, mild infection, and Cephalohematoma. However, there were no significant differences in terms of gender, BW, weight loss ≥ 10%, exclusive breastfeeding, ABO incompatibility, vitamin D levels, vitamin D status and maternal conditions. There was no evident difference in the prevalence of vitamin D deficiency, insufficiency, and sufficiency between NH and controls. But both the NH and control group have significant proportions of vitamin D deficiency individuals, and the proportion is a little higher in the NH group. In order to determine which characteristics influenced the severity of hyperbilirubinemia, the NH group was further classified as mild, moderate and severe group, and the reclassified population characteristics are shown in Supplementary Table 2. The mild, moderate and severe groups were comparable for GA, cephalohematoma and TSB. Beyond that, there was no significant difference in other aspects.

**Table 1:** Characteristics of the study population

Characteristics	Total (n = 336)	Control group (n = 149)	NH group (n = 187)	χ^2^/t/Z	*P*-Value
Neonatal characteristics					
Age, Median (IQR), day	4.50 (6.98)	6.00 ((8.00)	4.00 (5.75)	-4.63	**< 0.001**
Gender				2.99	0.084
Male, n (%)	189 (56.25%)	76 (51.01%)	113 (60.43%)		
Female, n (%)	147 (43.75%)	73 (48.99%)	74 (39.57%)		
Gestational age, Mean ± SD, day	273.28 ± 9.20	274.74 ± 8.55	272.12 ± 9.55	2.61	**0.009**
Birth weight, Median (IQR), g	3310.00 (607.50)	3370.00 (620.00)	3300.00 (610.00)	-1.34	0.179
Weight loss ≥ 10%, n (%)	14 (4.17%)	3 (2.01%)	11 (5.88%)	3.35	0.067
Breast feeding, n (%)	87 (25.89%)	41 (27.52%)	46 (24.60%)	0.368	0.544
Mild infection, n (%)	224 (66.67%)	108 (72.48%)	116 (62.03%)	4.08	**0.043**
Cephalohematoma, n (%)	56 (16.67%)	14 (9.40%)	42 (22.46%)	10.19	**0.001**
ABO incompatibility, n (%)	66 (19.64%)	29 (19.46%)	37 (19.79%)	0.01	0.941
Vitamin D, Median (IQR), ng/ml	9.82(7.06)	10.38(7.12)	9.48(7.56)	-1.79	0.074
Vitamin D status				4.55	0.103
Sufficiency, n (%)	28 (8.33%)	17 (11.41%)	11 (5.88%)		
Insufficiency, n (%)	37 (11.01%)	19 (12.75%)	18 (9.63%)		
Deficiency, n (%)	271 (80.66%)	113 (75.84%)	158 (84.49%)		
Maternal characteristics					
Age, Mean ± SD, years	28.97 ± 4.23	28.63 ± 4.18	29.24 ± 4.27	-1.31	0.190
Primigravida, n (%)	200 (59.52%)	89 (59.73%)	111 (59.36%)	0.01	0.945
Pregnancy Complications, n (%)	184 (54.76%)	79 (53.02%)	105 (56.15%)	0.33	0.567

### Vitamin D deficiency and genotypes

The relationship between the genotypes of the six SNPs and vitamin D deficiency and the relationship between NH and vitamin D deficiency are shown in Table 2. The study population was divided into the vitamin D deficiency group and non-vitamin D deficiency group based on whether the vitamin D level was lower than 15 ng/ml. Only rs12785878 (*NADSYN1/DHCR7*) and rs10877012 (*CYP27B1*) were significantly different in vitamin D levels. After adjusting for age, GA, mild infection, cephalohematoma, ABO incompatibility and the season, TT genotype at rs12785878 was protective factor for vitamin D deficiency. However, after adjusting for the potential confounding variables mentioned above, the rs10877012 TT genotype did not independently affect the risk for vitamin D deficiency, but the rs10877012 GT genotype was significantly different. It is worth mentioning that the relationship between the NH group and vitamin D status (in terms of deficiency, insufficiency and sufficiency) is not significant. However, we found a higher frequency of vitamin D deficiency in NH group after reclassifing the study population as vitamin D deficiency and non-vitamin D deficiency groups, whereas there was no significant difference after adjusting for potential confounding variables.

**Table 2:** Correlation between NH and vitamin D deficiency and correlation between vitamin D deficiency and its metabolic pathway genes polymorphisms

	Non-vitamin D deficiency (n = 65)	Vitamin D deficiency (n = 271)	Crude OR (95% CI)	*P*-Value	Adjusted OR (95% CI)	*P*-Value
Control group	36 (55.38%)	113 (41.70%)	1		1	
NH group	29 (44.62%)	158 (58.30%)	1.74 (1.01, 2.99)	**0.048**	1.18 (0.65, 2.14)	0.588
*NADSYN1/DHCR7* rs12785878						
GG	17(26.15%)	67 (24.72%)	1		1	
GT	30 (46.15%)	129 (47.60%)	0.67 (0.39, 1.16)	0.153	0.69 (0.39, 1.24)	0.218
TT	18 (27.70%)	75 (27.68%)	0.45 (0.25, 0.83)	**0.011**	0.45 (0.23, 0.86)	**0.016**
*GC* rs4588						
CC	38 (58.46%)	120 (44.28%)	1		1	
CA	22 (33.85%)	117 (43.17%)	0.77 (0.49, 1.22)	0.261	0.79 (0.49, 1.30)	0.359
AA	5 (7.69%)	34 (12.55%)	1.31 (0.64, 2.72)	0.460	1.22 (0.56, 2.65)	0.620
*GC* rs7041						
TT	24 (36.92%)	142 (52.40%)	1		1	
TG	30 (46.16%)	90 (33.21%)	0.93 (0.58, 1.49)	0.774	1.09 (0.65, 1.80)	0.750
GG	11 (16.92%)	39 (14.39%)	1.79 (0.92, 3.50)	0.086	1.55 (0.76, 3.15)	0.224
*CYP2R1* rs12794714						
CC	29 (44.61%)	108 (39.85%)	1		1	
CT	28 (43.08%)	113 (41.70%)	0.99 (0.62, 1.59)	0.979	0.97 (0.58, 1.61)	0.902
TT	8 (12.31%)	50 (18.45%)	1.06 (0.57, 1.97)	0.855	1.14 (0.59, 2.23)	0.692
*CYP24A1* rs17216707						
TT	56 (86.15%)	247 (91.14%)	1		1	
CT	9 (13.85)	24 (8.86%)	1.25 (0.60, 2.61)	0.547	1.19 (0.54, 2.61)	0.665
*CYP27B1* rs10877012						
GG	19 (29.23%)	56 (20.66%)	1		1	
GT	30 (46.15%)	130 (47.97%)	0.60 (0.34, 1.06)	0.076	0.53 (0.29, 0.98)	**0.042**
TT	16 (24.62%)	85 (31.37%)	0.49 (0.26, 0.90)	**0.023**	0.60 (0.31, 1.16)	0.131

Abbreviations: CI, confidence interval; OR, odds ratio. Adjusting for age, gestational age, mild infection, cephalohematoma, ABO incompatibility and the season

### The correlation between genotype and NH

The analysis of the genotypes, alleles and genetic model frequencies of six vitamin D metabolic pathway genes SNPs between the NH and control groups are shown in Table 3. The frequencies distribution of the six SNPs did not deviate from HWE. No homozygous mutation was detected at the rs17216707 locus in *CYP24A1* gene. For SNP rs12785878, the frequencies of TT genotype, T allele, dominant model, and recessive model were significantly lower in the NH group than in the control group. After adjusting for potential confounding variables, TT genotype, T allele and recessive model at rs12785878 were all protective factors for NH (0 < OR < 1, *P* < 0.05). The risk of NH in carriers of the rs12785878 TT genotype, T allele and recessive model were 0.45, 0.66 and 0.76 times that of the GG genotype, G allele and GG + GT genotypes, the adjusted ORs (and 95% CI) were 0.45 (0.23, 0.86), 0.66 (0.48, 0.92) and 0.76 (0.58, 0.98), respectively. For SNP rs10877012, the frequencies of TT genotype, T allele and dominant model were lower in the NH group than in the control group. After adjusting for potential confounding variables, GT genotype and dominant model at rs10877012 were all protective factors for NH (0 < OR < 1, *P* < 0.05). The risk of NH in carriers of the rs10877012 GT genotype and dominant model were 0.53 and 0.56 times that of the GG genotype, the adjusted ORs (and 95% CI) were 0.53 (0.29, 0.98) and 0.56 (0.31, 0.99), respectively.

**Table 3:** Correlation between NH and vitamin D metabolic pathway genes polymorphisms

		Control group (n = 149)	NH group (n = 187)	*P*_H−W_	Crude OR (95% CI)	P-Value	Adjusted OR (95% CI)	*P*-Value
*NADSYN1/DHCR7* rs12785878								
Genotype	GG	29 (19.46%)	55 (29.41%)	0.806	1		1	
	GT	70 (46.98%)	89 (47.59%)		0.67 (0.39, 1.16)	0.153	0.69 (0.39, 1.24)	0.218
	TT	50 (33.56%)	43 (23.00%)		0.45 (0.24, 0.83)	**0.011**	0.45 (0.23, 0.86)	**0.016**
Allele	G	128 (42.95%)	199 (53.21%)		1		1	
	T	170 (57.05%)	175 (46.79%)		0.66 (0.49, 0.90)	**0.008**	0.66 (0.48, 0.92)	**0.013**
Dominant model	GT + TT	120 (80.54%)	132 (70.59%)		0.58 (0.35, 0.97)	**0.038**	0.59 (0.34, 1.02)	0.059
	GG	29 (19.46%)	55 (29.41%)		1		1	
Recessive model	GG + GT	99 (66.44%)	144 (77.01%)		0.77 (0.60, 0.98)	**0.032**	0.76 (0.58, 0.98)	**0.036**
	TT	50 (33.56%)	43 (22.99%)		1		1	
*GC* rs4588								
Genotype	CC	67 (44.97%)	91 (48.66%)	0.782	1		1	
	CA	68 (45.64%)	71 (37.97%)		0.77 (0.49, 1.22)	0.261	0.79 (0.49, 1.30)	0.359
	AA	14 (9.839%)	25 (13.37%)		1.31(0.64, 2.72)	0.460	1.22 (0.56, 2.65)	0.620
Allele	C	202 (67.79%)	253 (67.65%)		1		1	
	A	96 (32.21%)	121 (32.35%)		1.00 (0.85, 1.18)	0.970	0.99 (0.83, 1.18)	0.950
Dominant model	CA + AA	82 (55.03%)	96 (51.33%)		0.86 (0.56, 1.33)	0.500	0.87 (0.55, 1.38)	0.554
	CC	67 (44.97%)	91 (48.66%)		1		1	
Recessive model	CC + CA	135 (90.60%)	162 (86.63%)		1.22 (0.86, 1.73)	0.261	1.16 (0.80, 1.69)	0.423
	AA	14 (9.40%)	25 (13.37%)		1		1	
*GC* rs7041								
Genotype	TT	76 (51.00%)	90 (48.13%)	0.054	1		1	
	TG	57 (38.26%)	63 (33.69%)		0.93 (0.58, 1.49)	0.774	1.09 (0.65, 1.80)	0.750
	GG	16 (10.74%)	34 (18.18%)		1.79 (0.92, 3.50)	0.086	1.55 (0.76, 3.15)	0.224
Allele	T	209 (70.13%)	243 (64.97%)		1		1	
	G	89 (29.87%)	131 (35.03%)		1.13 (0.96, 1.32)	0.157	1.11 (0.94, 1.33)	0.228
Dominant model	TG + GG	73 (48.99%)	97 (51.87%)		1.12 (0.73, 1.73)	0.600	1.20 (0.76, 1.91)	0.438
	TT	76 (51.01%)	90 (48.13%)		1		1	
Recessive model	TT + TG	133 (89.26%)	153 (81.82%)		1.36 (0.99, 1.87)	0.059	1.22 (0.87, 1.72)	0.240
	GG	16 (10.74%)	34 (18.18%)		1		1	
*CYP2R1* rs12794714								
Genotype	CC	61 (40.94%)	76 (40.64%)	0.343	1		1	
	CT	63 (42.28%)	78 (41.71%)		0.99 (0.62, 1.59)	0.979	0.97 (0.58, 1.61)	0.902
	TT	25 (16.78%)	33 (17.65%)		1.06 (0.57, 1.97)	0.855	1.14 (0.59, 2.23)	0.692
Allele	C	185 (62.08%)	230 (61.50%)		1		1	
	T	113 (37.92%)	144 (38.50%)		1.01 (0.87, 1.18)	0.877	1.03 (0.87, 1.21)	0.755
Dominant model	CT + TT	88 (59.06%)	111 (59.36%)		1.01 (0.65, 1.57)	0.956	1.02 (0.64, 1.63)	0.941
	CC	61 (40.94%)	76 (40.64%)		1		1	
Recessive model	CC + CT	124 (83.22%)	154 (82.35%)		1.03 (0.77, 1.37)	0.834	1.08 (0.79, 1.47)	0.634
	TT	25 (16.78%)	33 (17.65%)		1		1	
*CYP24A1* rs17216707								
Genotype	TT	136 (91.28%)	167 (89.30%)	0.802	1		1	
	CT	13 (8.72%)	20 (10.70%)		1.25 (0.60, 2.61)	0.547	1.19 (0.54, 2.62)	0.665
Allele	T	285 (95.64%)	354 (94.65%)		1		1	
	C	13 (4.36%)	20 (5.35%)		1.24 (0.61, 2.53)	0.55	1.18 (0.55, 2.54)	0.674
*CYP27B1* rs10877012								
Genotype	GG	25 (16.78%)	50 (26.74%)	0.880	1		1	
	GT	73 (49.00%)	87 (46.52%)		0.60 (0.34, 1.06)	0.076	0.53 (0.29, 0.98)	**0.042**
	TT	51 (34.22%)	50 (26.74%)		0.49 (0.26, 0.91)	**0.024**	0.60 (0.31, 1.16)	0.131
Allele	G	123 (41.28%)	187 (50.00%)		1		1	
	T	175 (58.72%)	187(50.00%)		0.84 (0.72, 0.98)	**0.024**	0.89 (0.75, 1.05)	0.162
Dominant model	GT + TT	124 (83.22%)	137 (73.26%)		0.55 (0.32, 0.95)	**0.031**	0.56 (0.31, 0.99)	**0.045**
	GG	25 (16.78%)	50 (26.74%)		1		1	
Recessive model	GG + GT	98 (65.77%)	137 (73.26%)		0.84 (0.66, 1.06)	0.138	0.96 (0.74, 1.24)	0.758
	TT	51 (34.23%)	50 (26.74%)		1		1	

Abbreviations: CI, confidence interval; OR, odds ratio. Adjusting for age, gestational age, mild infection, cephalohematoma, ABO incompatibility and the season

The genotypes, allele frequencies and genetic model of the six SNPs observed in mild, moderate and severe groups are shown in Table 4. The GT genotype and dominant model distribution of rs12785878 were significantly different among the mild, moderate and severe groups, and the carriers of the GT genotype and dominant model of rs12785878 had a higher risk of severe NH than the GG genotype carriers, the adjusted ORs (and 95% CI) were 2.43 (1.22, 4.86) and 1.97 (1.04, 3.73), respectively.

**Table 4:** Correlation between the severity of NH and vitamin D metabolic pathway genes polymorphisms

		Mild (n = 76)	Moderate (n = 93)	Severe (n = 18)	Crude OR (95% CI)	*P*-Value	Adjusted OR (95% CI)	*P*-Value
*NADSYN1/DHCR7* rs12785878								
Genotype	GG	15 (19.74%)	33 (35.48%)	7 (38.89%)	1		1	
	GT	45 (59.21%)	37 (39.79%)	7 (38.89%)	2.47 (1.27, 4.79)	**0.008**	2.43 (1.22, 4.86)	**0.012**
	TT	16 (21.05%)	23 (24.73%)	4 (22.22%)	1.51 (0.69, 3.28)	0.300	1.37 (0.61, 3.05)	0.445
Allele	G	75 (49.34%)	103 (55.38%)	21 (58.33%)	1		1	
	T	77 (50.66%)	83 (44.62%)	15 (41.67%)	1.29 (0.87, 1.91)	0.201	1.23 (0.82, 1.85)	0.312
Dominant model	GT + TT	61 (80.26%)	60 (64.52%)	11 (61.11%)	2.09 (1.12, 3.88)	**0.020**	1.97 (1.04, 3.73)	**0.037**
	GG	15 (19.74%)	33 (35.48%)	7 (38.89%)	1		1	
Recessive model	GG + GT	60 (78.95%)	70 (75.27%)	14 (77.78%)	0.87 (0.45, 1.68)	0.678	0.82 (0.41, 1.63)	0.570
	TT	16 (21.05%)	23 (24.73%)	4 (22.22%)	1		1	
*GC* rs4588								
Genotype	CC	40 (52.63%)	45 (48.39%)	6 (33.33%)	1		1	
	CA	26 (34.21%)	37 (39.78%)	8 (44.45%)	0.71 (0.39, 1.29)	0.257	0.71 (0.38, 1.33)	0.291
	AA	10 (13.16%)	11 (11.83%)	4 (22.22%)	0.71 (0.30, 1.66)	0.426	0.76 (0.31, 1.86)	0.551
Allele	C	106 (69.74%)	127 (68.28%)	20 (55.56%)	1		1	
	A	46 (30.26%)	59 (31.72%)	16 (44.44%)	0.78 (0.51, 1.18)	0.240	0.81 (0.52, 1.24)	0.330
Dominant model	CA + AA	36 (47.37%)	48 (51.61%)	12 (66.67%)	0.71 (0.41, 1.23)	0.220	0.73 (0.41, 1.29)	0.277
	CC	40 (52.63%)	45 (48.39%)	6 (33.33%)	1		1	
Recessive model	CC + CA	66 (86.84%)	82 (88.17%)	14 (77.78%)	0.82 (0.36, 1.85)	0.636	0.88 (0.38, 2.06)	0.770
	AA	10 (13.16%)	11 (11.83%)	4 (22.22%)	1		1	
*GC* rs7041								
Genotype	TT	41 (53.95%)	38 (40.86%)	11 (61.11%)	1		1	
	TG	26 (34.21%)	32 (34.41%)	5 (27.78%)	0.95 (0.51, 1.76)	0.863	0.85 (0.44, 1.63)	0.619
	GG	9 (11.84%)	23 (24.73%)	2 (11.11%)	0.62 (0.29, 1.33)	0.221	0.69 (0.31, 1.52)	0.355
Allele	T	108 (71.05%)	108 (58.06%)	27 (75.00%)	1		1	
	G	44 (28.95%)	78 (41.94%)	9 (25.00%)	0.77 (0.51, 1.16)	0.205	0.79 (0.52, 1.21)	0.285
Dominant model	TG + GG	35 (46.05%)	55 (59.14%)	7 (38.89%)	0.81 (0.47, 1.41)	0.460	0.78 (0.44, 1.39)	0.406
	TT	41 (53.95%)	38 (40.86%)	11 (61.11%)	1		1	
Recessive model	TT + TG	67 (88.16%)	70 (75.27%)	16 (88.89%)	0.63 (0.31, 1.31)	0.217	0.74 (0.35, 1.55)	0.421
	GG	9 (11.84%)	23 (24.73%)	2 (11.11%)	1		1	
*CYP2R1* rs12794714								
Genotype	CC	35 (46.05%)	35 (37.63%)	6 (33.33%)	1		1	
	CT	31 (40.79%)	37 (39.79%)	10 (55.56%)	0.72 (0.39, 1.33)	0.298	0.81 (0.43, 1.56)	0.535
	TT	10 (13.16%)	21 (22.58%)	2 (11.11%)	0.62 (0.28, 1.37)	0.241	0.54 (0.24, 1.24)	0.149
Allele	C	101 (66.45%)	107 (57.53%)	22 (61.11%)	1		1	
	T	51 (33.55%)	79 (42.47%)	14 (38.89%)	0.750 (0.50, 1.12)	0.162	0.72 (0.48, 1.10)	0.128
Dominant model	CT + TT	41 (53.95%)	58 (62.37%)	12 (66.67%)	0.69 (0.39, 1.21)	0.198	0.72 (0.39, 1.30)	0.271
	CC	35 (46.05%)	35 (37.63%)	6 (33.33%)	1		1	
Recessive model	CC + CT	66 (86.84%)	72 (77.42%)	16 (88.89%)	0.73 (0.35, 1.52)	0.402	0.60 (0.28, 1.29)	0.189
	TT	10 (13.16%)	21 (22.58%)	2 (11.11%)	1		1	
*CYP24A1* rs17216707								
Genotype	TT	70 (92.11%)	83 (89.25%)	14 (77.78%)	1		1	
	CT	6 (7.89%)	10 (10.75%)	4 (22.22%)	0.50 (0.20, 1.24)	0.134	0.53 (0.21, 1.35)	0.183
Allele	T	146 (96.05%)	176 (94.62%)	32 (88.89%)	1		1	
	C	6 (3.95%)	10 (5.38%)	4 (11.11%)	0.52 (0.22, 1.25)	0.144	0.55 (0.22, 1.37)	0.199
*CYP27B1* rs10877012								
Genotype	GG	19 (25.00%)	27 (29.03%)	4 (22.22%)	1		1	
	GT	35 (46.05%)	42 (45.16%)	10 (55.56%)	0.99 (0.51, 1.95)	0.990	1.01 (0.50, 2.06)	0.969
	TT	22 (28.95%)	24 (25.81%)	4 (22.22%)	1.22 (0.57, 2.60)	0.605	1.18 (0.54, 2.60)	0.677
Allele	G	73 (48.03%)	96 (51.61%)	18 (50.00%)	1		1	
	T	79 (51.97%)	90 (48.39%)	18 (50.00%)	1.11 (0.75, 1.66)	0.589	1.10 (0.73, 1.64)	0.659
Dominant model	GT + TT	57 (75.00%)	66 (70.97%)	14 (77.78%)	1.07 (0.58, 2.00)	0.823	1.08 (0.56, 2.07)	0.826
	GG	19 (25.00%)	27 (29.03%)	4 (22.22%)	1		1	
Recessive model	GG + GT	54 (71.05%)	69 (74.19%)	14 (77.78%)	1.22 (0.66, 2.29)	0.526	1.17 (0.61, 2.25)	0.632
	TT	22 (28.95%)	24 (25.81%)	4 (22.22%)	1		1	

Abbreviations: CI, confidence interval; OR, odds ratio. Adjusting for age, gestational age, mild infection, cephalohematoma, ABO incompatibility and the season

### Haplotype-based analysis results

LD was assessed between the two *GC* SNPs genotyped within our data by the Haploview software. As shown in Supplementary Fig. 1, D’ values = 1 indicated complete LD and D’>0.8 is strong LD as represented by a red-colored square. The SNPs of rs4588 and rs7041 were in complete LD. Haplotype association with NH and vitamin D deficiency susceptibility are shown in Table 5. AT haplotype was associated with vitamin D deficiency, while no haplotype showed significant differences between the NH and control groups.

**Table 5:** Haplotype association with NH and vitamin D deficiency susceptibility

Haplotype	NH group (freq)	Control group (freq)	Chi Square	*P*-Value	Non-vitamin D deficiency (freq)	Vitamin D deficiency (freq)	Chi Square	*P*-Value
CT	0.368	0.400	0.709	0.3997	0.379	0.395	0.118	0.7315
CG	0.308	0.278	0.737	0.3906	0.280	0.359	3.139	0.0765
AT	0.282	0.301	0.317	0.5737	0.311	0.205	5.751	**0.0165**
AG	0.042	0.021	2.374	0.1234	0.030	0.041	0.414	0.5199

SNP-IDs of haplotypes: rs4588, rs7041. freq, frequency

### SNP-SNP and SNP-vitamin D levels interaction models

GMDR was used to screen for the best interaction combination among the six SNPs of the vitamin D metabolic pathway genes and to uncover the effect of SNP-vitamin D levels interaction on NH risk (Table 6). We found no significant SNP-SNP and SNP-vitamin D levels interaction combinations after adjusting for age, GA, mild infection, cephalohematoma, ABO incompatibility and the season.

**Table 6:** SNP-SNP and SNP-vitamin D levels interaction models in NH obtained using the GMDR method

Model	Training Bal. Acc.	Testing Bal. Acc.	CVC	*P* ^a^
Gene-gene interaction				
rs10877012	0.5551	0.4886	5/10	0.623
rs12785878, rs10877012	0.6036	0.5376	10/10	0.623
rs12785878, rs10877012, rs12794714	0.6475	0.5210	6/10	0.0547

Bal. Acc., balanced accuracy; CVC, cross-validation consistency; OR, odds ratio; 95% CI, 95% confidence interval. ^a^The analyses were performed under logistic regression adjusted for age, gestational age, mild infection, cephalohematoma, ABO incompatibility and the season

## Discussion

NH is a global pediatric issue. Although the causes of NH are complex, the contribution of genetic factors to the pathogenesis of NH has received increasing attention. Our study showed that the proportion of vitamin D deficiency was higher in the NH group than in controls, and rs12785878 (*NADSYN1/DHCR7*) and rs10877012 (*CYP27B1*) are correlated with NH risk and vitamin D deficiency. In addition, we found that there are no relationships between vitamin D levels and the severity of NH, whereas GT heterozygotes and the dominant model of rs12785878 are two negative factors for severe NH. In this study, the correlation of serum vitamin D concentrations, vitamin D metabolic pathway genes polymorphisms and the risk of hyperbilirubinemia were investigated in Chinese neonates, to the best knowledge of the authors, this is the first study to analyze the relationship between the three. This will help offer new perspectives on the diagnosis and management of NH and assist in the identification of severe NH.

In this study, the median vitamin D level is 10.38 ng/ml in controls and 9.48 ng/ml in NH. The vitamin D level in cases was lower than that in controls, but the difference is not significant. Furthermore, neonates with NH had a higher rate of vitamin D deficiency compared with the controls, but there was no significant difference after adjusting for potential confounding variables. The relationship between vitamin D and NH is not well studied so far, the mechanism remains to be discovered, and the possible explanations for their relationship are as follows [10, 26]. Firstly, vitamin D can reduce erythropoietin levels in the blood, which can decrease the lysis of red blood cells and bilirubin production; Secondly, vitamin D can slow down oxidative stress, cell and tissue damage [27], which may help prevent the accumulation of bilirubin levels induced by red blood cell oxidative damage; Thirdly, the liver is the important organ for the metabolism of both bilirubin and vitamin D, so, their metabolic processes may have potential interaction. Notably, many studies have indicated that vitamin D plays an important part in neurotrophic regulation and neuroprotection, neurotransmission and neuroplasticity[28]. Therefore, we speculate that vitamin D deficiency is associated with bilirubin encephalopathy caused by severe NH. However, vitamin D levels are not associated with the severity of NH in our study. Similar to our study, another study suggested that a significant negative relationship was also observed between vitamin D levels and bilirubin levels in the newborns (*P* < 0.001). Meanwhile, there was no significant difference in vitamin D levels among mild, moderate and severe NH[26]. However, a study from Iran revealed a lack of a correlation between vitamin D levels and NH[8]. A recent meta-analysis showed that vitamin D level of the NH group was significantly lower than that in the healthy control group, and its subgroup analysis based on the bilirubin level of the study indicated that vitamin D levels might cause differences in the degree of NH[10]. Therefore, we speculated that low vitamin D level is a risk factor for NH and may also be linked to severe NH. However, vitamin D levels are influenced by a variety of factors. Although we adjusted for some potential confounding variables, future studies should refine groups and increase the sample size to explore the relationship between vitamin D levels and NH.

Vitamin D exists in different forms in the body with several enzymes being involved in their metabolism. 7-dehydrocholesterol reductase encoded by the *DHCR7* converts 7-hydroxycholesterol (the precursor of vitamin D) to cholesterol. Vitamin D requires two hydroxylation steps to become an active hormone, 25-hydroxylation in the liver and 1α-hydroxylation in the kidney which is catalyzed by the enzyme encoded by the *CYP2R1* and *CYP27B1*, respectively. The vitamin D and its metabolites bind to vitamin D-binding protein encoded by *GC* gene and are transported to target tissues and organs, its action is exerted by binding to the nuclear vitamin D receptor, and the degradation of these vitamin D metabolites is initiated by 24-hydroxylase encoded *CYP24A1* [29]. A study revealed that the two SNPs in *GC* (rs222020, rs2298849), four SNPs in *CYP2R1* (rs10741657, rs10766197, rs12794714 and rs1562902) and two SNPs in *NADSYN1/DHCR7* (rs3829251, rs12785878) were associated with plasma 25(OH)D concentrations in northeastern Han Chinese children[30]. Likewise, previous studies also demonstrated that *GC* gene (rs7041 and rs4588) and their haplotype were key factors of vitamin D levels in infants and toddlers [31, 32]. Findings from the present study suggested that *NADSYN1/DHCR7* rs12785878 TT genotypes and *CYP27B1* rs10877012 GT genotypes were associated with decreased risk of vitamin D deficiency. Furthermore, we established haplotypes for *GC* gene from different combinations of selected two SNPs, and the AT haplotype was associated with vitamin D deficiency. The possible explanation is that the amino acid substitutions at these sites influence the O-glycosylation of the vitamin D binding protein, affect the vitamin D binding protein half-life and then decrease vitamin D levels[32].

The vitamin D metabolic pathway genes are shown to associate with NH susceptibility by different analyses in this study, including the use of a single SNP model, SNP-SNP interactions and haplotype analysis. In our study, the TT genotype, T allele and recessive model of the rs12785878 SNP were associated with decreased risk of NH, whereas the GT genotype and dominant model of the rs12785878 SNP were two significant risk factors for developing severe NH. And GT genotype and dominant model of rs10877012 were protective factors for NH. The rs10877012 is located in the promotor region of the *CYP27B1* gene, which might impact transcription and translation processes[33]. The rs12785878 is located in 8 kilobases upstream from the transcription initiation site of *DHCR7* and in an intron of *NADSYN1*[34]. To date, there are no functional studies of the rs12785878, so it remains unclear by which mechanism this SNP influences vitamin D levels and NH risk. The etiology of NH is complex, and to identify the complex biological relationships leading to NH, we attempted to understand the epistatic phenomenon involved in NH etiology through SNP-SNP and SNP- vitamin D levels interaction analysis. Both multivariate logistic regression as well as epistasis analysis yielded no significant results(*P* > 0.05). However, rs12785878-rs10877012-rs12794714 SNP combination may be potentially associated with the risk of NH (the testing balanced accuracy was 52.10% and cross-validation consistency was 6/10, *P* = 0.0547). There were no differences in the frequencies of CT, CG, AT and AG haplotypes for *GC* gene between the case and control groups. This study is the first to analyze the relationship between vitamin D metabolic pathway genes polymorphisms and the risk of hyperbilirubinemia in Chinese neonates, which could provide new clues for further studies on the genetic variant of NH.

Our study also had some limitations. There is a possibility of selection bias since this was a hospital-based retrospective study. The sample size is relatively small. The information of mother’s vitamin D supplementation during pregnancy was not available. Therefore, further studies involved in various populations with larger sample size are required to validate our findings.

## Conclusions

The present study confirms that the proportion of vitamin D deficiency was higher in the NH group than in controls, and rs12785878 (*NADSYN1/DHCR7*) and rs10877012 (*CYP27B1*) are correlated with NH risk and vitamin D deficiency, which supports the biologic explanation of a connection between the vitamin D metabolic pathway genes polymorphisms and NH risk. Furthermore, rs12785878 was associated with severe NH risk. The current study enhanced the cognition of NH at the genetic level in China and provided a new target for the prevention, diagnosis and treatment of NH.

## Electronic supplementary material

Below is the link to the electronic supplementary material.

Supplementary Material 1

## Data Availability

The database used and analyzed in the present study is not publicly available as its information may compromise the participants’ privacy and consent involved in the research, but are available from the corresponding author on reasonable request.

## List of abbreviations

BW: birth weight
G6PD: Glucose-6-Phosphate Dehydrogenase
GA: gestational age
GMDR: Generalized multifactor dimensionality reduction
GWAS: genome-wide association studies
HRM: High resolution melting
HWE: Hardy-Weinberg equilibrium
LC-MS/MS: liquid chromatography-tandem mass spectrometry
LD: Linkage disequilibrium
NH: Neonatal hyperbilirubinemia
Rh: Rhesus Macacus
SNP: single nucleotide polymorphisms
TSB: total serum bilirubin
