# Supplementary material for: Vitamin D metabolic pathway genes polymorphisms and vitamin D levels in association with neonatal hyperbilirubinemia in China: a single-center retrospective cohort study
Source: BMC Pediatr. 2023 May 31;23:275. doi: 10.1186/s12887-023-04086-y (PMC10230706; doi:10.1186/s12887-023-04086-y)

Supplementary Table 1. Sequences of PCR and HGM primers.

| Gene | SNPs | Sequence of PCR primers (5’-3’) | Sequence of HGM primers (5’-3’) |
| --- | --- | --- | --- |
| *NADSYN1/DHCR7* | rs12785878 | F: GAATACCACCTTCAAATAGGGC  R: AGCATCTGGGCAACATCAG | F: CCTAAGTGCCAAGGGATCTA  R: AGGCTCACGAGACGATCAG |
| *GC* | rs4588 | F: CTCGAAGAGGCATGTTTCACT  R: CTGCCATGTTAAGTGGAGGGT | F: AAGCAAAATTGCCTGATGCC  R: CTGCCATGTTAAGTGGAGGGT |
|  | rs7041 | F: CTCGAAGAGGCATGTTTCACT  R: CTGCCATGTTAAGTGGAGGGT | F: TTTCAGACTGGCAGAGCGAC  R: AGCTTTGCCAGTTCCGTGG |
| *CYP2R1* | rs12794714 | F: AAGGTAGTTGTCCCGAAGGG  R: GGCCATAAGTCCAACCAGGAA | F: ATCGGCAACATCTATTCCC  R: TACACCTGGCTCTGCTTTC |
| *CYP24A1* | rs17216707 | F: TGCTTCCCTGACCAAATC  R: TTCATCACCTCCACGACTC | F: TCCCTGACCAAATCCTGAG  R: CCGTATGTTTAGGTGAGAATCG |
| *CYP27B1* | rs10877012 | F: AGCTGACTCGGTCTCCTCTG  R: ACCATCCTCCTGTCCTCTCC | F: TAGGCAACAGAGAGAGGGC  R: TGAGGGAGTAAGGAGCAGAG |

Supplementary Table 2. Characteristics of the NH

| Characteristics | Mild (n=76) | Moderate (n=93) | Severe (n=18) | χ2/t/Z | *P*-Value |
| --- | --- | --- | --- | --- | --- |
| Neonatal characteristics |  |  |  |  |  |
| Age, Median (IQR), day | 3.00 (6.95) | 4.00 (5.15) | 3.05 (6.84) | 1.285 | 0.526 |
| Gender |  |  |  | 0.244 | 0.885 |
| Male, n (%) | 47 (61.84%) | 56 (60.22%) | 10 (55.56%) |  |  |
| Female, n (%) | 29 (38.16%) | 37 (39.78%) | 8 (44.44%) |  |  |
| Gestational age, Mean ±SD, day | 274.99±8.43 | 270.28±9.41 | 269.56±11.90 | 6.13 | 0.003^**^ |
| Birth weight, Median (IQR), g | 3305.00 (587.50) | 3200.00 (725.00) | 3375.00 (575.00) | 1.97 | 0.373 |
| Weight loss≥10%, n (%) | 2 (2.63%) | 7 (7.53%) | 2 (11.11%) | 2.79 | 0.274 |
| Breast feeding, n (%) | 17 (22.37%) | 23 (24.73%) | 6 (33.33%) | 0.95 | 0.623 |
| Mild infection, n (%) | 53 (69.74%) | 51 (54.84%) | 12 (66.67%) | 4.12 | 0.127 |
| Cephalohematoma, n (%) | 10 (13.16%) | 22 (23.66%) | 10 (55.56%) | 15.17 | 0.001^**^ |
| ABO incompatibility, n (%) | 10 (13.16%) | 23 (24.73%) | 4 (22.22%) | 3.60 | 0.165 |
| TSB, Median (IQR), μmol/L | 237.60(147.18) | 294.80(106.20) | 380.30(155.05) | 54.11 | <0.001^***^ |
| Vitamin D, Median (IQR), ng/ml | 9.29(7.71) | 9.71(6.16) | 8.96(9.68) | 0.01 | 0.996 |
| Vitamin D status |  |  |  | 6.74 | 0.120 |
| Sufficiency, n (%) | 7 (9.21%) | 2 (2.15%) | 2 (11.11%) |  |  |
| Insufficiency, n (%) | 5 (6.58%) | 12 (12.90%) | 1 (5.56%) |  |  |
| Deficiency, n (%) | 64 (84.21%) | 79 (84.95%) | 15 (83.33%) |  |  |
| Maternal characteristics |  |  |  |  |  |
| Age, Mean ±SD, years | 28.93±4.38 | 29.48±4.41 | 29.28±2.91 | 0.35 | 0.709 |
| Primigravida, n (%) | 43 (56.58%) | 59 (63.44%) | 9 (50.00%) | 1.54 | 0.463 |
| Pregnancy Complications, n (%) | 38 (50.00%) | 57 (61.29%) | 10 (55.56%) | 2.17 | 0.338 |

^*^, P<0.05; ^**^, P<0.01; ^***^, P<0.001. TSB: total serum bilirubin.

Supplementary Fig. 1 The patterns of linkage disequilibrium in the *GC* gene, with their |D’|.


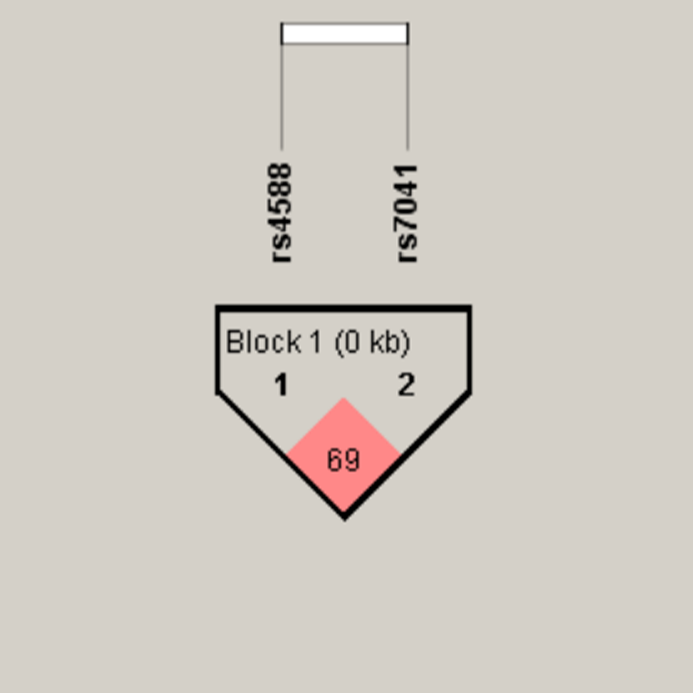

Supplement: Supplementary file 1 — Supplementary Material 1 [file 12887_2023_4086_MOESM1_ESM.docx]
